# Supplementary material for: Impact of six-month COVID-19 travel moratorium on Plasmodium falciparum prevalence on Bioko Island, Equatorial Guinea
Source: Nat Commun. 2024 Sep 27;15:8285. doi: 10.1038/s41467-024-52638-2 (PMC11436818; doi:10.1038/s41467-024-52638-2)
Supplement: Supplementary file 1 — Supplementary Information [file 41467_2024_52638_MOESM1_ESM.pdf]

Supplementary Information for: Impact of six-month COVID-19 travel moratorium on *Plasmodium falciparum* prevalence on Bioko Island, Equatorial Guinea

Contents

Supplementary Table 1. Comparison of select individual and household characteristics by year and travel area. 2
Supplementary Table 2. Design-based linear regression model outputs looking at malaria prevalence in high and low travel areas in 2019 and 2020, unadjusted and adjusted for relevant covariates.. 4
Supplementary Table 3. Exponentiated design-based logistic regression model outputs looking at malaria infection in high and low travel areas in 2019 and 2020, unadjusted and adjusted for relevant covariates 5
Supplementary Table 4. *Plasmodium falciparum (Pf)* prevalence in 2019 and 2020 for three enumeration areas with known land use changes during the study period, as well as prevalence in all low travel areas combined in each year. 6
Supplementary Table 5. Prevalence differences (PD) comparing high and low travel areas by year.. 7
Supplementary Table 6. Design-based linear regression model outputs model outputs for an analysis of *Pf* prevalence by year and travel group that included a community level care seeking parameter (prop_treat), using responses from 12,128 individuals. 8
Supplementary Table 7. Design-based linear regression model outputs for an adjusted analysis of *Pf* prevalence by year and travel group, stratified by stratum or urban/rural designation of the enumeration units included in the analysis. 8
Supplementary Table 8. General linear regression model outputs of interaction terms for a model of *Pf* prevalence by year and travel group for 2015 to 2019 MIS data. 9
Supplementary Table 9. *Plasmodium falciparum* (*Pf)* prevalence by year and travel area in individuals who indicated traveling to the mainland in the past 8-weeks compared to those who did not report recent travel.. 9

Supplementary Figure 1. Comparison of odds of infection in different years and travel areas (high or low) on Bioko Island [panels A-E].. 10
Supplementary Figure 2. Areas in urban Malabo, Bioko Island, Equatorial Guinea where *Anopheles* larvae were identified by vector control teams. 11

Supplementary Table 1. Comparison of select individual and household characteristics by year and travel area. Characteristics were selected *a priori* because of their relationship with malaria transmission. Parameters bolded and shaded in gray are those that were adjusted for in the final model. The total individuals (ind) and households (hh) from each group are presented.

	Low Travel EAs		High Travel EAs		All	
**Parameter**	**2019**	**2020**	**2019**	**2020**	**2019**	**2020**
**Individual Parameters (sample size)**	n ind = 2355	n ind = 1958	n ind = 4536	n ind = 4346	n ind = 6891	n ind = 6304
Age in years	25.1 (21.6); 0.1-110	26.5 (21.8); 0.1-90	21.4 (18.0); 0.1-110	21.9 (18.1); 0.1-95	21.8 (18.1); 0.1-110	22.0 (18.1); 0.1-95
Female	1209 (51.3%)	994 (50.8%)	2426 (53.5%)	2392 (55.0%)	3635 (52.7%)	3386 (53.7%)
Permanent resident of household	2124 (90.2%)	--	4269 (94.6%)	--	6518 (94.6%)	--
Is employed	549 (38.7%)	493 (42.7%)	1047 (38.5%)	956 (38.8%)	1596 (38.6%)	1449 (40.1%)
Travelled off-island in past 8-weeks	56 (2.4%)	5 (0.3%)	458 (10.1%)	55 (1.3%)	514 (7.5%)	60 (1.0%)
Average trips off island	0.0 (0.2); 0-3	0.0 (0.1); 0-2	0.1 (0.4); 0-5	0.0 (0.2); 0-2	0.1 (0.4); 0-5	0.0 (0.1); 0-2
**Travelled within island in past 8-weeks**	442 (18.8%)	244 (13.3%)	597 (13.2%)	378 (9.1%)	1039 (15.1%)	622 (10.3%)
Slept in household previous night	2262 (96.1%)	1789 (97.1%)	4404 (97.1%)	4071 (97.3%)	6666 (96.7%)	5860 (97.3%)
Slept under a bed net previous night	1008 (44.6%)	736 (41.2%)	1598 (36.3%)	1418 (34.9%)	2606 (39.1%)	2154 (36.8%)
**Go inside before 7pm**	511 (22.5%)	432 (25.1%)	1244 (29.2%)	1377 (35.4%)	1755 (26.9%)	1809 (32.2%)
Go to sleep before 9pm	618 (28.6%)	495 (29.6%)	636 (16.0%)	611 (16.1%)	1256 (20.4%)	1106 (20.3%)
Sick in past two-weeks	262 (11.2%)	166 (9.0%)	462 (10.3%)	314 (7.6%)	736 (10.7%)	457 (7.6%)
Sought care for illness	136 (52.1%)	75 (45.2%)	322 (68.4%)	170 (58.4%)	458 (62.6%)	245 (53.6%)
**Household Parameters (sample size)**	n hh = 825	n hh = 813	n hh = 1696	n hh = 1674	n hh = 2521	n hh = 2487
Household size	3.9 (2.9); 1-16	3.3 (2.4); 1-18	4.2 (2.8); 1-16	3.9 (2.6); 1-16	4.1 (2.8); 1-16	3.7 (2.6); 1-18
Number of visitors in house	0.1 (0.2); 0-1	0.1 (0.2); 0-1	0.0 (0.2); 0-1	0.1 (0.2); 0-1	0.0 (0.2); 0-1	0.1 (0.2); 0-1
Has the following item:						
Sofa	569 (69.1%)	600 (73.8%)	1425 (84.0%)	1426 (85.2%)	1994 (79.1%)	2026 (81.5%)
Table	634 (76.8%)	664 (81.7%)	1355 (79.9%)	1392 (83.2%)	1989 (78.9%)	2056 (82.7%)
Armoire	365 (44.2%)	358 (44.0%)	1108 (65.4%)	1000 (59.7%)	1473 (58.5%)	1358 (54.6%)
**Air conditioning**	59 (7.2%)	65 (8.0%)	555 (32.7%)	644 (38.5%)	614 (24.4%)	709 (28.5%)
Stove	417 (50.5%)	442 (54.4%)	1375 (81.1%)	1418 (84.7%)	1792 (71.1%)	1860 (74.8%)
Washing Machine	145 (17.6%)	149 (18.3%)	549 (32.4%)	626 (37.4%)	694 (27.5%)	775 (31.2%)
Car	129 (15.6%)	127 (15.6%)	567 (33.5%)	568 (33.9%)	696 (27.6%)	695 (27.9%)
**Household sprayed in past 6-months**	**358 (46.9%)**	**274 (35.1%)**	**418 (28.3%)**	**897 (57.2%)**	**776 (34.6%)**	**1171 (49.8%)**
Own bed nets	181 (46.2%)	196 (43.9%)	259 (26.1%)	341 (34.6%)	440 (31.8%)	537 (37.5%)

	Low Travel EAs		High Travel EAs		All	
**Parameter**	**2019**	**2020**	**2019**	**2020**	**2019**	**2020**
Housing type:						
Social housing	11 (1.3%)	13 (1.6%)	200 (11.8%)	161 (9.6%)	211 (8.4%)	174 (7.0%)
Barracks	125 (15.2%)	152 (18.7%)	594 (35.0%)	617 (36.9%)	719 (28.5%)	769 (30.9%)
Apartment	19 (2.3%)	14 (1.7%)	136 (8.0%)	114 (6.8%)	155 (6.1%)	128 (5.1%)
Other	670 (81.2%)	634 (78.0%)	766 (45.2%)	782 (46.7%)	1436 (57.0%)	1416 (56.9%)

Supplementary Table 2. Design-based linear regression model outputs looking at malaria prevalence in high and low travel areas in 2019 and 2020, unadjusted and adjusted for relevant covariates. The *Intercept* is the modeled prevalence in low travel areas in 2019. The *trav* parameter is the modeled difference in prevalence comparing high travel to low travel areas in 2019. The *year* parameter is the modeled difference in prevalence comparing 2020 to 2019 in low travel areas. The *trav:year* parameter, the interaction between time and year, is the DID term that shows the modeled difference in difference of the change in prevalence in 2020 to 2019 in high travel areas compared to the change in prevalence in 2020 to 2019 in low travel areas. There were 6891 observations in 2019 and 6304 observations in 2020.

	Unadjusted model		Adjusted model	
Parameter	Estimate	95% CI	Estimate	95% CI
(Intercept)	0.073	(0.045, 0.102)	0.064	(0.033, 0.095)
trav	0.063	(0.032, 0.094)	0.077	(0.047, 0.107)
year	0.055	(0.008, 0.102)	0.058	(0.004, 0.111)
trav:year	-0.074	(-0.123, -0.025)	-0.092	(-0.147, -0.037)
inbefore7	-	-	-0.032	(-0.046, -0.017)
spry_prec	-	-	0.058	(0.014, 0.101)
aircon	-	-	-0.024	(-0.048, -0.001)
travelledisland	-	-	0.010	(-0.014, 0.034)

Supplementary Table 3. Exponentiated design-based logistic regression model outputs looking at malaria infection in high and low travel areas in 2019 and 2020, unadjusted and adjusted for relevant covariates Odds ratios (OR) and 95% confidence intervals (CI) of infection shown for the main model, and when removing three sites known to have large land use changes between the two study years. Main model includes 12,128 observations, and sensitivity analysis includes 11,447 observations.

	Main Model		Sensitivity Analysis (removed 3 EAs with land use changes)	
Comparison	Unadjusted model OR (95% CI)	Adjusted Model* OR (95% CI)	Unadjusted model OR (95% CI)	Adjusted Model* OR (95% CI)
High travel areas 2020/High travel 2019	0.84 (0.75,0.96)	0.72 (0.6,0.86)	0.84 (0.74,0.96)	0.72 (0.61,0.85)
Low travel 2020/Low travel 2019	1.86 (1.22,2.84)	1.92 (1.18,3.12)	1.64 (1,2.67)	1.62 (0.97,2.71)
High travel 2020/Low travel 2019	1.69 (1.08,2.63)	1.65 (1.14,2.39)	2.06 (1.25,3.4)	1.91 (1.19,3.07)
High travel 2020/Low travel 2020	0.91 (0.53,1.54)	0.86 (0.52,1.43)	1.26 (0.84,1.87)	1.18 (0.83,1.69)
High travel 2019/Low travel 2019	2.0 (1.30,3.05)	2.29 (1.53,3.44)	2.44 (1.5,3.95)	2.66 (1.66,4.26)
High travel areas 2020/high travel areas 2019 to Low travel areas 2020/low travel areas 2019	0.45 (0.29, 0.71)	0.38 (0.22,0.63)	0.52 (0.31, 0.86)	0.44 (0.26,0.76)

* Adjusted for spray coverage, going indoors before 7pm, within island travel and air-conditioning

Supplementary Table 4. Survey mean estimated *Plasmodium falciparum (Pf)* prevalence in 2019 and 2020 for three enumeration areas with known land use changes during the study period, as well as prevalence in all low travel areas combined in each year. N value is total individuals tested in the area.

	2019		2020	
Community	n	Prevalence	n	Prevalence
All low travel areas	2268	7.50%	1721	12.80%
Basupu	103	17.5%	77	45.5%
Baloeri	166	22.9%	180	32.8%
Baticopo	127	7.9%	102	7.8%

Supplementary Table 5. Prevalence differences (PD) and 95% confidence interval (CI) comparing high and low travel areas by year. Estimates were obtained using linear combinations of the design-based linear regression model outputs for an analysis of *Pf* prevalence by year and travel group. Results show for the main model, and when removing three sites know to have large land use changes between the two study years. Main model includes 12,128 observations, and sensitivity analysis includes 11,447 observations.

	**Main Model**		**Sensitivity Analysis (removed 3 EAs with land use changes)**	
**Comparison**	**Unadjusted model PD (95% CI)**	**Adjusted Model* PD (95% CI)**	**Unadjusted model PD (95% CI)**	**Adjusted Model* PD (95% CI)**
High travel areas 2020-High travel 2019	-1.9 (-3.2,-0.5)	-3.4 (-5.1,-1.8)	-1.9 (-3.2,-0.5)	-3.4 (-5,-1.8)
Low travel 2020-Low travel 2019	5.5 (0.9,10.1)	5.8 (0.5,11)	3.5 (0.1,6.9)	3.4 (-0.1,6.9)
High travel 2020-Low travel 2019	4.4 (1.1,7.7)	4.3 (1.5,7)	5.7 (2.5,8.9)	5 (1.9,8.2)
High travel 2020-Low travel 2020	-1.1 (-7,4.8)	-1.5 (-7.2,4.2)	2.2 (-1.4,5.8)	1.6 (-1.6,4.9)
High travel 2019-Low travel 2019	6.3 (3.3,9.4)	7.7 (4.7,10.6)	7.6 (4.6,10.5)	8.4 (5.5,11.4)
High travel areas 2020-high travel areas 2019) - (Low travel areas 2020-low travel areas 2019)	***-7.4 (-12.1,-2.6)***	***-9.2 (-14.5,-3.9)***	***-5.4 (-9,-1.7)***	***-6.8 (-10.5,-3.1)***

Adjusted for spray coverage, going indoors before 7pm, within island travel and air-conditioning

Supplementary Table 6. Design-based linear regression model outputs of estimate and 95% confidence interval (CI) for an analysis of *Pf* prevalence by year and travel group that included a community level care seeking parameter (prop_treat), using responses from 12,128 individuals. The *Intercept* is the modeled prevalence in low travel areas in 2019. The *trav* parameter is the modeled difference in prevalence comparing high travel to low travel areas in 2019. The *year* parameter is the modeled difference in prevalence comparing 2020 to 2019 in low travel areas. The *trav:year* parameter, the interaction between time and year, is the DID term that shows the modeled difference in difference of the change in prevalence in 2020 to 2019 in high travel areas compared to the change in prevalence in 2020 to 2019 in low travel areas. *Prop_treat* was calculated as the proportion of individuals who were sick in the past two weeks that indicated seeking care of any kind. In low travel areas, 262 individuals reported being sick in 2019 and 166 reported being sick in 2020. In high travel areas, 474 individuals reported being sick in 2019 and 291 reported being sick in 2020.

Model parameter	estimate	95% CI
(Intercept)	0.039	(0,0.08)
trav	0.070	(0.04,0.1)
year	0.061	(0.01,0.11)
inbefore7	-0.032	(-0.05,-0.02)
spry_perc	0.059	(0.02,0.1)
aircon	-0.026	(-0.05,0)
travelledisland	0.011	(-0.01,0.03)
prop_treat	0.047	(-0.02,0.11)
trav:year	-0.093	(-0.15,-0.04)

Supplementary Table 7. Design-based linear regression model outputs for an adjusted analysis of *Pf* prevalence by year and travel group, stratified by stratum or urban/rural designation of the enumeration units included in the analysis. Model estimate, lower confidence limit (lcl) and upper confidence limit (ucl) and p-value of the estimate from regression model are presented. Of note, the *trav* parameter is the modeled difference in prevalence comparing high travel to low travel areas in 2019. The *year* parameter is the modeled difference in prevalence comparing 2020 to 2019 in low travel areas. The *trav:year* parameter, the interaction between time and year, is the DID term that shows the modeled difference in difference of the change in prevalence in 2020 to 2019 in high travel areas compared to the change in prevalence in 2020 to 2019 in low travel areas

	Stratum 1/Rural n= 3103				Stratum 2/Urban n=9025			
	Estimate	lcl	ucl	p-value	est	lcl	ucl	p-value
(Intercept)	0.057	0.019	0.094	0.006	0.065	0.027	0.104	0.002
trav	0.123	0.023	0.224	0.019	0.078	0.042	0.115	0.000
year	0.062	-0.003	0.128	0.061	0.055	-0.009	0.119	0.089
inbefore7	-0.032	-0.073	0.008	0.106	-0.031	-0.047	-0.016	0.000
spry_perc	0.107	0.015	0.200	0.026	0.041	-0.004	0.086	0.074
aircon	0.022	-0.114	0.157	0.737	-0.029	-0.054	-0.004	0.023
travelledisland	0.000	-0.028	0.029	0.998	0.010	-0.017	0.037	0.445
trav.year	-0.104	-0.195	-0.012	0.029	-0.083	-0.148	-0.018	0.014

Supplementary Table 8. General linear regression model outputs of interaction terms for a model of *Plasmodium falciparum* prevalence by year and travel group for 2015 to 2019 MIS data. The interaction terms provide an estimate in the difference between prevalence in high and low travel areas in the given year, compared to the difference between these two groups in 2015 (baseline). If trends are parallel, we would expect the effect sizes for these comparative differences to be close to zero and non-significant in each year. The total number of individuals included in the calculation for each year is as follows: 2015: high (n=5889), low (n=3560); 2016: high (n=5097), low(n=2799); 2017: high (n=5050), low(n=2642); 2018: high (n=4861), low (n=2647); 2019: high (n=4532), low(n=2354); 2020: high (n=4346), low (n=1957).

Measure	Effect Size	95% CI	p-value
Difference in prevalence between high and low travel areas 2015	7.98%	(4.48,11.48)	0.000
Difference in prevalence between high and low travel areas in 2016 compared to difference in 2015	-0.48%	(-2.36,1.4)	0.616
Difference in prevalence between high and low travel areas in 2017 compared to difference in 2015	-2.70%	(-4.6,-0.79)	***0.006***
Difference in prevalence between high and low travel areas in 2018 compared to difference in 2015	-0.11%	(-2.02,1.81)	0.914
Difference in prevalence between high and low travel areas in 2019 compared to difference in 2015	0.54%	(-1.47,2.54)	0.599

Supplementary Table 9. *Plasmodium falciparum* (*Pf)* prevalence by year and travel area in individuals who indicated traveling to the mainland in the past 8-weeks compared to those who did not report recent travel. Prevalence and confidence interval (CI) estimated using *svymean* in R.

		Overall		High Travel Areas		Low Travel Areas	
Year	Traveled on Island	n	*Pf* prevalence (95% CI)	n	*Pf* prevalence (95% CI)	n	*Pf* prevalence (95% CI)
2019	No	5839	13.3% (12.2, 14.4)	3926	13.9% (12.5, 15.2)	1913	7.2% (4.3, 10.0)
	Yes	1039	13.5% (11.5, 15.6)	597	12.8% (10.1, 15.6)	442	9.3% (4.2, 14.6)
2020	No	5395	11.8% (10.5, 13.1)	3798	11.6% (9.8, 13.5)	1597	12.4% (7.3, 17.6)
	Yes	622	13.1% (10.6, 15.4)	378	13.9% (10.6, 15.4)	244	13.8% (3.1, 24.5)

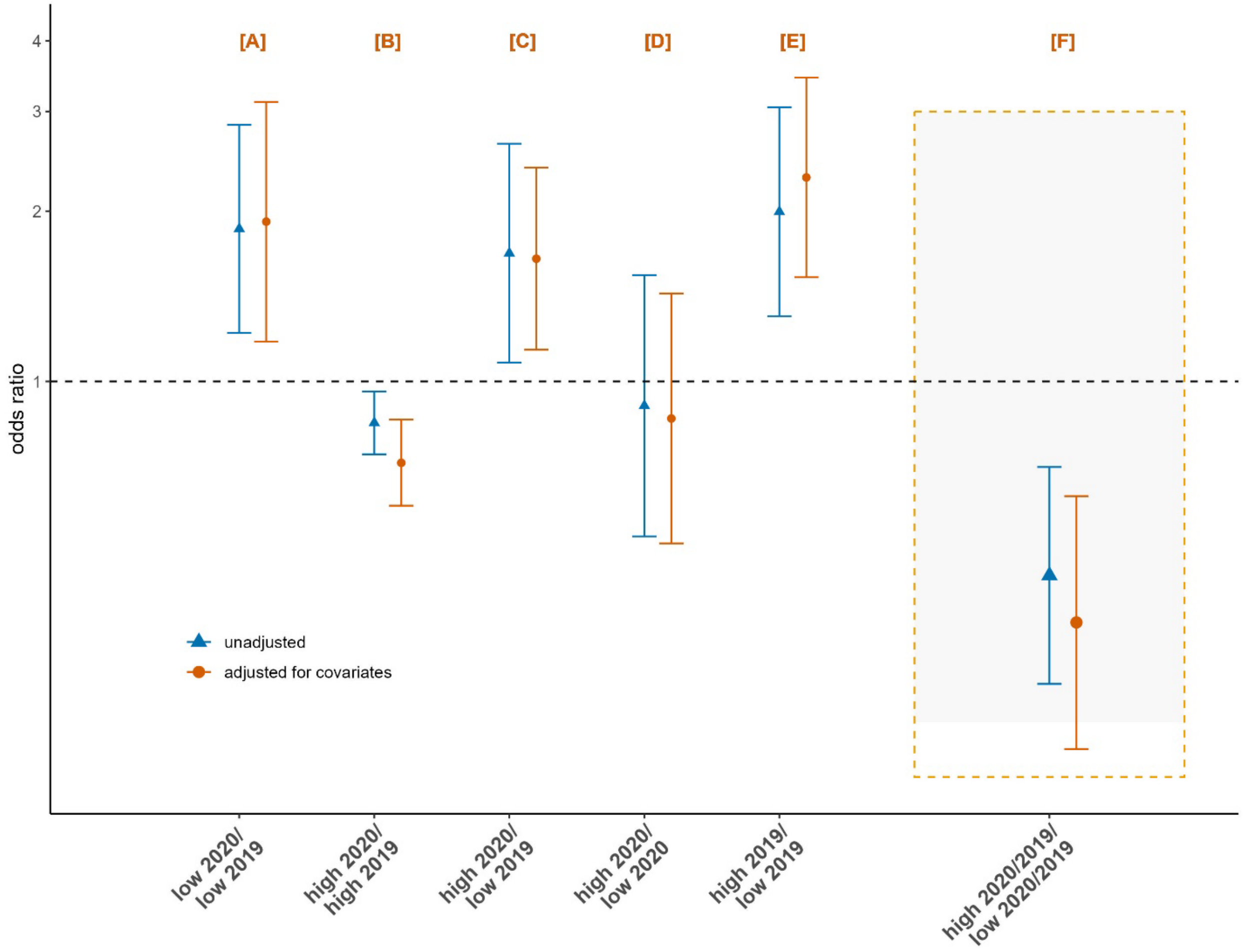

Supplementary Figure 1. Comparison of odds of infection in different years and travel areas (high or low) on Bioko Island [panels A-E]. Adjusted odds ratios (orange circles) are from models adjusted for spray coverage and whether an individual went inside before 7PM. The ratio of ratios comparing the change in odds in high travel areas between 2019 and 2020 relative to the change in low areas is shown in the gray box [panel F].

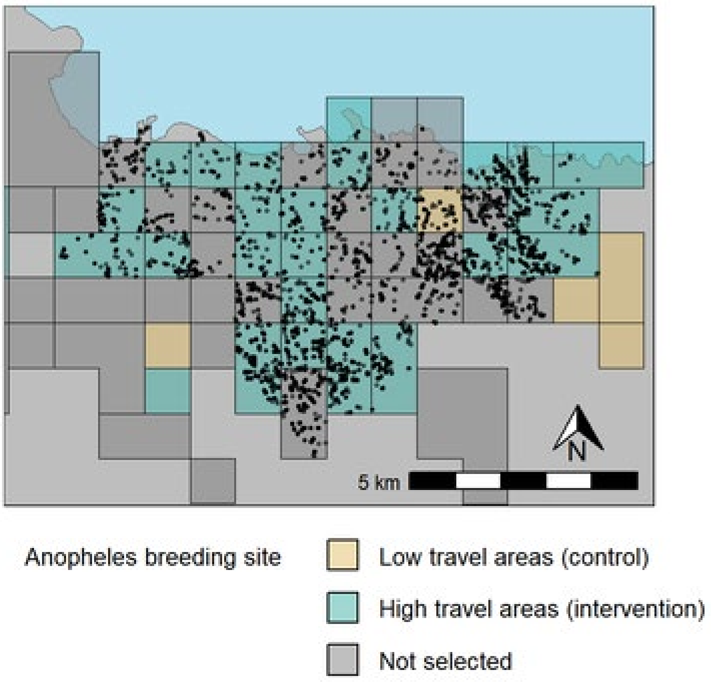

Supplementary Figure 2. Areas in urban Malabo, Bioko Island, Equatorial Guinea where *Anopheles* larvae were identified by vector control teams.
