# Supplementary material for: Impact of six-month COVID-19 travel moratorium on Plasmodium falciparum prevalence on Bioko Island, Equatorial Guinea
Source: Nat Commun. 2024 Sep 27;15:8285. doi: 10.1038/s41467-024-52638-2 (PMC11436818; doi:10.1038/s41467-024-52638-2)
Supplement: Supplementary file 3 — Reporting Summary [file 41467_2024_52638_MOESM3_ESM.pdf]

nature portfolio

Corresponding author(s): Dianna Hergott (dhergott@uw.edu)

Last updated by author(s): Sep 5, 2024

Reporting Summary

Nature Portfolio wishes to improve the reproducibility of the work that we publish. This form provides structure for consistency and transparency in reporting. For further information on Nature Portfolio policies, see our Editorial Policies and the Editorial Policy Checklist.

Statistics

For all statistical analyses, confirm that the following items are present in the figure legend, table legend, main text, or Methods section.

n/a	Confirmed	
☐	☒	The exact sample size (*n*) for each experimental group/condition, given as a discrete number and unit of measurement
☐	☒	A statement on whether measurements were taken from distinct samples or whether the same sample was measured repeatedly
☐	☒	The statistical test(s) used AND whether they are one- or two-sided<br>*Only common tests should be described solely by name; describe more complex techniques in the Methods section.*
☐	☒	A description of all covariates tested
☐	☒	A description of any assumptions or corrections, such as tests of normality and adjustment for multiple comparisons
☐	☒	A full description of the statistical parameters including central tendency (e.g. means) or other basic estimates (e.g. regression coefficient) AND variation (e.g. standard deviation) or associated estimates of uncertainty (e.g. confidence intervals)
☐	☒	For null hypothesis testing, the test statistic (e.g. *F*, *t*, *r*) with confidence intervals, effect sizes, degrees of freedom and *P* value noted<br>*Give P values as exact values whenever suitable.*
☒	☐	For Bayesian analysis, information on the choice of priors and Markov chain Monte Carlo settings
☐	☒	For hierarchical and complex designs, identification of the appropriate level for tests and full reporting of outcomes
☒	☐	Estimates of effect sizes (e.g. Cohen's *d*, Pearson's *r*), indicating how they were calculated

Our web collection on statistics for biologists contains articles on many of the points above.

Software and code

Policy information about availability of computer code

Data collection	Data was collected through a structured survey, coded with ODKCollect and managed on an in-house Android application for database maintenance. Field workers captured responses on a tablet, and data was uploaded to a central server at the end of each day.
Data analysis	All data analysis was conducted within R statistical software (v3.6.2). Travel prevalence estimates were derived using the R-INLA package using previously published methods (Guerra. 2019. Nat Comms). Cleaning and manipulation of variables was done using base R and dplyr (v1.0.6). Analyses on the survey data were done using the survey package in R (v4.1-1). Code has been provided on GitHub (https://github.com/d-hergott/DID-public)

For manuscripts utilizing custom algorithms or software that are central to the research but not yet described in published literature, software must be made available to editors and reviewers. We strongly encourage code deposition in a community repository (e.g. GitHub). See the Nature Portfolio guidelines for submitting code & software for further information.

Data

Policy information about availability of data

All manuscripts must include a data availability statement. This statement should provide the following information, where applicable:

- Accession codes, unique identifiers, or web links for publicly available datasets
- A description of any restrictions on data availability
- For clinical datasets or third party data, please ensure that the statement adheres to our policy

The data used for the analyses are available on GitHub (https://github.com/d-hergott/DID-public).

Research involving human participants, their data, or biological material

Policy information about studies with human participants or human data. See also policy information about sex, gender (identity/presentation), and sexual orientation and race, ethnicity and racism.

Reporting on sex and gender	We do not perform any analyses disaggregated by sex in this publication. The proportion of surveyed individuals who are female is presented in supplementary Table 1. Sex is collected through self-report in the demographic section of the survey. Options include male and female.
Reporting on race, ethnicity, or other socially relevant groupings	Ethnicity was not utilized in our analyses. Most respondents in our study group are from the same ethnic group (Fang).<br><br>Respondents were classified by the travel prevalence of their community. This information was obtained through self-reported travel history of survey respondents from 2015-2018 (yearly surveys). Participants are asked if they traveled outside of island and spent the night in the past 8 weeks. If they respond yes, they are further probed for the area of travel. Travel prevalence was based on the self-report of travel to the mainland of Equatorial Guinea.
Population characteristics	Participants were sampled from the population of Bioko Island, Equatorial Guinea. Included in this analysis are those who resided in historically high travel and low travel enumeration areas. All present and consenting members of the household were tested and surveyed. Mean age of the study population was 21.8 years, with mean age slightly higher in low travel areas (25.1) compared to high travel areas (21.4). High travel areas were more likely to be in the urban areas of Bioko Island, where low travel areas were mostly in more rural areas.
Recruitment	Households were randomly selected through an independent statistician. Selected households were then approached by the study team and asked if they wanted to participate in the survey. All present and consenting household members were tested and surveyed in the selected households. Field work is conducted Monday-Saturday during working hours. Therefore, the sample may be over represented by non-working, non-traveling adults who are not present during the survey. This was more likely in 2019 when there were no travel restrictions.
Ethics oversight	Prevalence and travel information came from the Malaria Indicator Survey. The MIS is approved by the Ministry of Health and Social Welfare of Equatorial Guinea. This analysis was determined to not involve human subjects by the University of Washington Human Subjects Review Board (STUDY00012460) and approved by the National Malaria Control Program (NMCP) of Equatorial Guinea. Informed consent was provided by all adults in selected households and the head of household provided consent for all children under the age of 18.

Note that full information on the approval of the study protocol must also be provided in the manuscript.

Field-specific reporting

Please select the one below that is the best fit for your research. If you are not sure, read the appropriate sections before making your selection.

☐ Life sciences ☒ Behavioural & social sciences ☐ Ecological, evolutionary & environmental sciences

For a reference copy of the document with all sections, see nature.com/documents/nr-reporting-summary-flat.pdf

Behavioural & social sciences study design

All studies must disclose on these points even when the disclosure is negative.

Study description	This is a quantitative analysis using data from two cross-sectional surveys, employing a difference in differences regression.
Research sample	The sampling units for the MIS are geographically defined enumeration areas (EAs); under this scheme all households on Bioko Island, Equatorial Guinea, were eligible for selection into the survey through a stratified, single-cluster survey design. This analysis utilized data from individuals living in the areas in the highest and lowest quartiles of historical travel history to the mainland of Equatorial Guinea. Of the approximately 75,000 households in the study area, 4,669 were selected for the 2019 survey and 4,188 were selected for the 2020 survey. Of those, 2338 and 2109 households were included in this analysis for each year. Individuals selected for the analysis were similar to those not selected. Mean age in selected sample was 21.8 years, and 20.6 in those not selected. 47% of the selected sample were male, which was the same as the not selected sample. The MIS is designed to be representative of the entire population. We chose to only include those in the highest and lowest travel stratum to maximize the

	ability to evaluate the possible effect of importation of malaria by selecting areas thought to have little importation and those thought to have the greatest amount.
Sampling strategy	EAs were classified into two strata based on population density and estimated local residual transmission. Stratum 1 includes EAs with generally lower population density and generally higher LRT, representing mostly rural populations, and stratum 2 includes EAs with generally higher population density and generally lower LRT, representing mostly urban populations. To select the sample for the MIS, within each EA, a simple random sample of households was taken using specified sampling fractions for each stratum: 24% for stratum 1, and 4.8% in stratum 2. For this analysis, we further reduced the research sample to only include respondents who lived in EAs determined to be "high travel" or "low travel". EAs in the middle two quartiles of travel distribution were excluded. No sample size calculation was done prior to selecting high and low travel areas, as we wanted to ensure that the selection of high/low groups was based on probability of importation more than achieving a sample size. Once the sample was selected, we ran a calculation to determine a minimum detectable difference in differences value from the sample we had. Roughly, we have 80% power and 95% confidence to detect a difference of ~5% between groups.
Data collection	Survey responses were collected through a structured, interviewer-administered questionnaire. The survey is coded in ODKCollect, and interviewers recorded responses into an electronic form on an Android tablet. Information on malaria infection was obtained through testing with a malaria CareStart RDT. Results of the RDT were recorded in the electronic form. This analysis was performed retrospectively, so the survey team collecting information was unaware of the study hypothesis.
Timing	Data collection occurred during August and September of 2019 and 2020.
Data exclusions	Data from enumeration areas that were not classified in the highest or lowest quartile of travel prevalence distribution were excluded from the analysis. Additionally, for the final analysis, we only included data from individuals who had complete data for all adjustment factors. As such, 1067 individuals were excluded from the analysis. There were no apparent differences between those excluded and included, and missingness was assumed to be random.
Non-participation	Non-participation by selected households is not formally maintained in a database. But, anecdotal evidence and experience suggests the number is very low, below 1% of selected households. Within households that were surveyed, about 20% of registered individuals were not present during the survey in 2020, and 15% of those present refused the RDT testing. Males were more likely than females to be absent from the household, and refusal was balanced between sexes. In 2019, 26% of registered individuals in participating households were absent during RDT testing, and 10% of those present refused RDT testing. Reasons for declining RDT testing are not formally recorded.
Randomization	No randomization in this study. Information was put into groups based on travel prevalence.

Reporting for specific materials, systems and methods

We require information from authors about some types of materials, experimental systems and methods used in many studies. Here, indicate whether each material, system or method listed is relevant to your study. If you are not sure if a list item applies to your research, read the appropriate section before selecting a response.

Materials & experimental systems

n/a	Involved in the study	
☒	☐	Antibodies
☒	☐	Eukaryotic cell lines
☒	☐	Palaeontology and archaeology
☒	☐	Animals and other organisms
☒	☐	Clinical data
☒	☐	Dual use research of concern
☒	☐	Plants

Methods

n/a	Involved in the study	
☒	☐	ChIP-seq
☒	☐	Flow cytometry
☒	☐	MRI-based neuroimaging

Plants

Seed stocks	*Report on the source of all seed stocks or other plant material used. If applicable, state the seed stock centre and catalogue number. If plant specimens were collected from the field, describe the collection location, date and sampling procedures.*
Novel plant genotypes	*Describe the methods by which all novel plant genotypes were produced. This includes those generated by transgenic approaches, gene editing, chemical/radiation-based mutagenesis and hybridization. For transgenic lines, describe the transformation method, the number of independent lines analyzed and the generation upon which experiments were performed. For gene-edited lines, describe the editor used, the endogenous sequence targeted for editing, the targeting guide RNA sequence (if applicable) and how the editor was applied.*
Authentication	*Describe any authentication procedures for each seed stock used or novel genotype generated. Describe any experiments used to assess the effect of a mutation and, where applicable, how potential secondary effects (e.g. second site T-DNA insertions, mosiacism, off-target gene editing) were examined.*
